# Next-Generation Sequencing Identifies Polyunsaturated Fatty Acid Responsive Genes in the Juvenile Rat Cerebellum

**DOI:** 10.3390/nu11020407

**Published:** 2019-02-15

**Authors:** Aaron A. Mehus, Aaron M. Dickey, Timothy P. L. Smith, Kathleen M. Yeater, Matthew J. Picklo

**Affiliations:** 1 USDA-ARS Grand Forks Human Nutrition Research Center, Grand Forks, ND 58203, USA; Matthew.Picklo@ARS.USDA.GOV; 2 USDA-ARS U.S. Meat Animal Research Center, Clay Center, NE 68933, USA; Aaron.Dickey@ARS.USDA.GOV (A.M.D.); Tim.Smith@ARS.USDA.GOV (T.P.L.S.); 3 USDA-ARS, Plains Area, Fort Collins, CO 80526, USA; Kathleen.Yeater@ARS.USDA.GOV

**Keywords:** juvenile, cerebellum, *n*-3 PUFA, next-generation sequencing, gene expression, brain development

## Abstract

Dietary *n*-3 polyunsaturated fatty acids (PUFA) influence postnatal brain growth and development. However, little data exist regarding the impacts of dietary *n*-3 PUFA in juvenile animals post weaning, which is a time of rapid growth. We tested the hypothesis that depleting dietary *n*-3 PUFA would result in modifications to the cerebellar transcriptome of juvenile rats. To test this hypothesis, three week old male rats (an age that roughly corresponds to an 11 month old child in brain development) were fed diets containing either soybean oil (SO) providing 1.1% energy from α-linolenic acid (ALA; 18:3*n*-3; ALA-sufficient) or corn oil (CO) providing 0.13% energy from ALA (ALA-deficient) for four weeks. Fatty acids (FAs) in the cerebellum were analyzed and revealed a 4-fold increase in n-6 docosapentaenoic acid (DPA; 22:5*n*-6), increases in arachidonic acid (AA; 20:4*n*-6) and docosatetraenoic acid (DTA; 22:4*n*-6), but no decrease in docosahexaenoic acid (DHA; 22:6*n*-3), in animals fed CO versus SO. Transcript abundance was then characterized to identify differentially expressed genes (DEGs) between the two diets. Upper quartile (UQ) scaling and transcripts per million (TPM) data normalization identified 100 and 107 DEGs, respectively. Comparison of DEGs from the two normalization methods identified 70 genes that overlapped, with 90% having abundance differences less than 2-fold. *Nr4a3*, a transcriptional activator that plays roles in neuroprotection and learning, was elevated over 2-fold from the CO diet. These data indicate that expression of *Nr4a3* in the juvenile rat cerebellum is responsive to dietary *n*-3 PUFA, but additional studies are needed clarify the neurodevelopmental relationships between *n*-3 PUFA and *Nr4a3* and the resulting impacts.

## 1. Introduction

Childhood undernutrition is endemic throughout the world. Nutritional deficiency can severely impact neurodevelopment, and several lines of evidence indicate the importance of dietary long-chain polyunsaturated fatty acids (LCPUFA) in proper brain development. Within the brain, docosahexaenoic acid (DHA, 22:6*n*-3) and arachidonic acid (AA, 20:4*n*-6) are the most abundant LCPUFA. In humans, brain accumulation of LCPUFA begins prenatally and continues until the 2nd postnatal year as reviewed in Janssen & Kiliaan 2014 [1]. During this period of infant neurodevelopment, LCPUFAs incorporate into cellular membranes, regulate gene expression, and facilitate neurogenesis [2,3]. Postnatal availability of LCPUFA is crucial for myelination of nerve fibers and neurite outgrowth [4,5,6,7]. Childhood deficiencies in LCPUFA are associated with cognitive, behavioral, and visual impairments in children and adolescents [1]. 

The rat cerebellum is primarily developed and remodeled postnatally; similarly, human cerebellar development carries into the first postnatal years [8,9,10,11]. In rat cerebellum, non-neuronal cells dynamically change their numbers by postnatal day 25 and approximately 150 million neurons are added within the first three postnatal months [9]. The cerebellum has long been recognized for regulating motor coordination but is also involved with higher cognitive function [12]. Early rat studies demonstrated that the developing cerebellum is particularly susceptible to undernutrition [13,14].

DHA is obtained directly from the diet or is produced indirectly through its precursor α-linolenic acid (ALA, 18:3*n*-3). Similarly, if AA is not obtained through dietary sources, it may be synthesized from its parent fatty acid linoleic acid (LA, 18:2*n*-6). Although less abundant than DHA or AA, brain docosapentaenoic acid (DPA*n*-6, 22:5*n*-6), resulting from the elongation and desaturation of AA, is linked to roles in brain health and function. Elevated content of DPA*n*-6 is considered a biomarker of *n*-3 PUFA deficiency in animal models [15]. The exact physiological role of DPA*n*-6 within the brain is not entirely clear, but increased levels of DPA*n*-6 are associated with deficits in learning and behavior [16]. 

Dietary LCPUFA content alters gene expression throughout the body in a tissue-specific manner [17]. LCPUFA directly bind and activate transcription factors that modulate expression of target genes [18]. For example, peroxisome proliferator-activated receptors (PPARs) are ligand-dependent transcription factors that bind specific *n*-3 and *n*-6 PUFAs and regulate gene expression [19]. Maternal *n*-3 PUFA deficient diets lead to elevated postnatal brain D2 (dopamine) receptors and D2 receptor mRNA levels in rats [20,21]. Genes important in the processes of synaptic plasticity and cognition are modified in the brains of rats fed PUFA-enriched diets with the expression of transthyretin (*Ttr*) being reduced and α-synuclein (*Snca*) and calmodulins (*Calm1-3*) being increased [18,22].

Previous means of studying global LCPUFA-dependent differences in the transcriptome of the rodent brain have relied on RNA or cDNA microarray technology that analyzed a fraction of the genome [22,23,24,25]. Microarrays offer high-throughput expression analysis for thousands of genes between experimental conditions; although, the results are largely dependent on the selection/quality of probes used on the chip, along with other limitations [26]. Next-generation sequencing (NGS) or RNA-sequencing (RNA-seq) is a powerful alternative to microarray technology and is becoming more cost effective. NGS offers a global approach to measure differentially expressed genes (DEGs) that is not dependent on probes, and also displays greater sensitivity and dynamic range of quantification than microarrays [26,27].

We have estimated that a three week old rat pup corresponds to an 11 month old child that has reached 70% maximum brain weight, and where myelination is still occurring within the corpus callosum and cerebellum by using an online model (http://www.translatingtime.net/) to compare brain development across species [28]. In this study, we sought to identify the degree to which dietary *n*-3 PUFA deficiency modifies gene expression within the cerebellum of young rats given the neurodevelopmental importance of LCPUFA in postnatal brain health. We tested the hypothesis that a diet deficient in *n*-3 PUFA would result in modifications to the juvenile cerebellar transcriptome by characterizing the fatty acid profile and transcript abundance in the cerebellum of rats with variable levels of *n*-3 PUFA in their diets. A subset of transcripts that appeared to be differentially abundant between dietary classes from analysis of the RNAseq data, were validated with Quantitative PCR (qPCR) using RNA from two separate cohorts of animals. Cerebellar FA analysis demonstrated a 4-fold increase in DPA*n*-6, and slight increases of AA and docosatetraenoic acid (DTA; 22:4*n*-6) in the cerebellum of CO-fed animals. *Nr4a3* is an immediate early gene (IEG) that does not require de novo protein synthesis for its expression, therefore, can be activated and transcribed rapidly. *Nr4a3* encodes a transcriptional activator that plays roles in neuroprotection and learning and memory formation. *Nr4a3* was confirmed in both cohorts of animals to be elevated over 2-fold in the cerebellum from the CO diet.

## 2. Materials and Methods 

### 2.1. Reagents

Hexane, 2-propanol, acetyl chloride, and butylated hydroxytoluene (BHT) were from Sigma-Aldrich, St. Louis, MO, USA. Nonadecanoic acid and fatty acid methyl ester standards were purchased from Nu-Chek Prep. Inc., Elysian, MN, USA. 

### 2.2. Animals

All experiments were performed in accordance with the NIH guidelines for the use of live animals and were approved by the Institutional Animal Care and Use Committee of the USDA Agricultural Research Service, Grand Forks Human Nutrition Research Center. The animal experimentation and diets have been previously described and represent the same experimental conditions performed two separate times [29,30]. Male Sprague–Dawley rats (21 days old) were purchased from Envigo (Madison, WI, USA). Two days after arrival, rats were equally distributed, based on body mass, into two diet groups (*n* = 10/group): diets containing soybean oil (SO) with replete α-linolenic acid (ALA; 18:3*n*-3) with ad libitum (AL) intake and corn oil (CO) with AL intake. Oils constituted 16% fat energy. All diets were based on the AIN93G formula [31]. The SO diets had linoleic acid (LA; 18:2*n*–6) and ALA at 8.6% energy and 1.1% energy, respectively, whereas the CO diet had LA and ALA at 8.8% energy and 0.13% energy, respectively. The fatty acid composition of the oils were described previously with SO at ~6.6 mol% ALA and CO at ~0.8 mol% ALA [29].

Vitamin-free casein (Envigo) was used as a protein source. CO and SO were purchased from Dyets Inc (Bethlehem, PA, USA). All diets contained AIN93 mineral mix (Dyets Inc.) and AIN93G vitamin mix (Envigo). Caloric content of the diets was determined by bomb calorimetry as described previously [32]. Food consumption was measured daily, and fresh food was provided daily. Body mass was measured weekly. Body composition was determined by whole-body MRI (EchoMRI-700; Echo Medical Systems LLC, Houston, TX, USA) at the beginning and end of the study. 

Rats remained in their dietary groups for four weeks. At the end of the experiment, rats were fasted overnight. Rats were killed by an overdose of anesthetic (xylazine 13 mg/kg and ketamine 100 mg/kg) followed by exsanguination. The cerebellar region was dissected from the brain. Tissues were snap-frozen in liquid nitrogen and stored at −80 °C. Tissues were pulverized using a BioPulverizer (Biospec Products, Inc., Bartlesville, OK, USA) at liquid nitrogen temperature. Pulverized samples were stored at −80 °C until further use.

### 2.3. cDNA Library Preparation

RNA was isolated from pulverized tissue (~20 mg) using a QIAcube system (Qiagen, Valencia, CA, USA). RNA samples were treated with DNase I (New England Biolabs, Ipswich, MA, USA) for 10 min at 37 °C followed by the addition of EDTA and a 10 min incubation at 70 °C to stop the reaction. RNeasy MinElute spin columns (Qiagen) were used to concentrate the RNA before rRNA depletion with RiboZero Gold human/mouse/rat (Illumina, San Diego, CA, USA) and a subsequent MinElute concentration. The RNA quality number (RQN) was assessed using a Fragment Analyzer automated capillary electrophoresis system (Advanced Analytical, Ankeny, IA, USA). Table A1 displays the RQNs for this study; the average RQN was 8.0. Library construction was completed with a ScriptSeq V2 Library Preparation Kit and ScriptSeq Index Primers (Epicentre, Madison, WI, USA) according to the protocol of the manufacturer. Final library profiles were validated using a Fragment Analyzer (Advanced Analytical Technology, Ames, IA, USA) and quantified by qPCR with NEBNext (New England Biolabs, Ipswich, MA, USA). The 20 libraries were combined in equimolar amounts to a pool and loaded at 1.8 pM for sequencing with a 150 cycle high output paired end kit on a NextSeq 500 platform (Illumina, San Diego, CA, USA) in a single run to preclude sequence run effects on the output. No libraries from other sources were included in the run. After the run, fastq files for libraries based on the index sequences were created using bcl2fastq v2.17 (Illumina, San Diego, CA, USA), with approximately 5% of reads unclassified. 

### 2.4. NGS Mapping and Data Normalization

The sequencing data included 160 raw read files. Yield from the 20 cDNA libraries ranged from 11.0–54.9 million paired-end reads. Reads were adapter-trimmed using the BBDuk tool with options ktrim = *r k* = 23 mink = 11 hdist = 1 tpe = t tbo = t. The rat genome (Rnor_6.0) was indexed in STAR [33] with a NCBI annotated splice junction file (GCF_000001895.5_Rnor_6.0_genomic.gff.gz) that was converted from gff to gtf format using the cufflinks gffread tool [34]. Trimmed reads were mapped to the rat genome with STAR options—outFilterIntronMotifs = RemoveNoncanonical—quantMode = TranscriptomeSAM, GeneCounts—outSAMtype = BAM SortedByCoordinate. The raw count matrix was created using column 3 of the GeneCounts output files following developer recommendations for stranded paired-end sequence data. NGS data are displayed in Table 3. Genes in the annotation file were identified as protein coding if they possessed a CDS annotation. Gene lengths were calculated in R by sorting exons by gene in GenomicFeatures summing lengths across all annotated exons for a gene while accounting for overlap between exons [35]. Upon manuscript acceptance, raw sequence data will be made publicly available through the NCBI sequence read archive (accession # SRP144196). Trimmed data and assemblies are available from the authors upon request.

Counts were summarized by gene, resulting in 37,795 initial observations/rows. Prior to normalization, the summarized counts were filtered by removing rows with excessively missing, zero values, or low-value counts (2 or less) and then normalized using UQ scaling. After filtering, 17,109 genes remained for normalization (Table S1). Normalization of the sequencing data using Upper Quartile Scaling [36] was performed in JMP/Genomics 8.1 (SAS Institute, Inc., Cary, NC, USA), and normalized values were log2 transformed. For TPM normalization, the CDS only transcripts (22,926) were subset from the initial observations. The CDS only observations were filtered as described above. After filtering, 14,487 observations remained for normalization (Table S2). Normalization of the CDS only sequencing data using TPM (transcripts per million) calculation [37] was performed in SAS 9.4 (SAS Institute, Inc., Cary, NC, USA), and normalized values were log2 transformed.

### 2.5. qPCR Analysis

RNA isolation and qPCR analysis have previously been described [38]. RNA was isolated from approximately 20mg of pulverized tissue by using the QIAcube system (Qiagen, Valencia, CA, USA) following the manufacturer’s protocols. The quantity and quality of RNA were determined on a NanoDrop 8000 spectrophotometer (Thermo Fisher Scientific, Wilmington, DE, USA). RNA (1 μg) was converted to cDNA using the high-capacity cDNA reverse transcription kit (Applied Biosystem, Inc., Carlsbad, CA, USA) following the manufacturer’s protocols. Real time PCR reactions contained the following: 1X SYBR Green Master Mix (Applied Biosystem, Inc., Carlsbad, CA, USA), 300 nM of forward and reverse primers, and 40 ng cDNA. Real Time PCR was performed on a Quantstudio 12k flex Real-Time PCR System (Applied Biosystem, Inc., Carlsbad, CA, USA). Real-time qPCR primers were designed and purchased from IDT (Coralville, IA, USA). Expression of candidate genes was normalized to β-actin expression levels and evaluated in comparison to the control (SO diet) using the ΔΔCT method. Primer pairs for gene expression are presented in Table A2.

### 2.6. FA Analyses

Lipids were extracted using hexane:isopropanol [39]. A powdered sample of tissue (∼50 mg) was extracted twice into 3.6 mL of hexane/2-propanol (3:2 *v*/*v*) with BHT (50 μM; Sigma-Aldrich, St. Louis, MO, USA) added to limit lipid peroxidation. Samples were homogenized using a PRO200 Bio-Gen Series homogenizer (PRO Scientific Inc., Oxford, CT, USA) and then centrifuged at 2000× *g* for 10 min. The organic phase was removed, dried under nitrogen, and dissolved in 1 mL of hexane/2-propanol (3:2 *v*/*v*) containing 5% water and 50 μM BHT and stored at −80 °C under nitrogen. The FA content of the organic extract was determined by fatty acid methyl ester (FAME) analysis using a Thermo Trace-1310 equipped with a TriPlus RSH Autosampler, (Thermo Fisher Scientific, Waltham, MA, USA) and a Supelco SP-2560 capillary column (75 m, 0.18 mm ID, 0.14 µm film thickness) as previously described [38]. Fatty acid methyl esters were prepared with the use of acetyl chloride [38]. 

### 2.7. Statistical Analysis

For NGS data, statistical analysis was performed on all normalized, log2-transformed data using ANOVA with differences between the two diet treatment groups as part of the ANOVA analysis for RNA-Seq in JMP Genomics (SAS Institute, Inc., Cary, NC, USA). The significance threshold was based upon the default Multiple Testing Method, Benjamini-Hochberg’s FDR adjustment [40] and is set at 3.550 for the UQ scaling normalized data, and 3.430 for the TPM normalized data. 

Statistical analysis of qPCR and FA data were done with GraphPad Prism version 7.00 for Windows (GraphPad Software, La Jolla, CA, USA, www.graphpad.com). Standard deviations of the mean were reported. Statistical significance was assessed with two-tailed Student’s *t*-test with *p* ≤ 0.05. 

## 3. Results

### 3.1. Energy Intake and Body Composition

There were no differences in energy intake (data not shown), brain mass, or brain-to-body ratio between the rats consuming the SO or CO diets (Table 1). Within the 1st cohort, the liver and body mass of rats consuming the CO diets were slightly higher than rats consuming the SO diet but was unchanged in the 2nd cohort.

### 3.2. Cerebellar Fatty Acid Analysis

Table 2 displays the cerebellar FA analysis of the rats used for generating the RNA-seq data. There were increases in AA, DTA, and *n*-6 DPA, but no decrease in DHA in the cerebellum of CO-fed animals. The 4-fold increase in *n*-6 DPA within the cerebellum of CO-fed rats was the most prominent FA modification between the groups.

### 3.3. Next-generation Sequencing of Rat Cerebellum

Table 3 outlines the sequencing data. There was a five-fold span in range for the raw paired reads (11,037,221 to 54,876,507) and the reads that uniquely mapped to the rat genome (8,945,862 to 45,878,644) but were not significantly different (*p* = 0.3226 and *p* = 0.2454, respectively) between the two diet treatments. Approximately, 80% of the reads mapped uniquely to the rat genome. On average this correlated to just over 21,000 annotated genes identified with a range of 19,354 to 23,180.

### 3.4. Upper Quartile (UQ) Scaling Normalization

**Figure 1:**
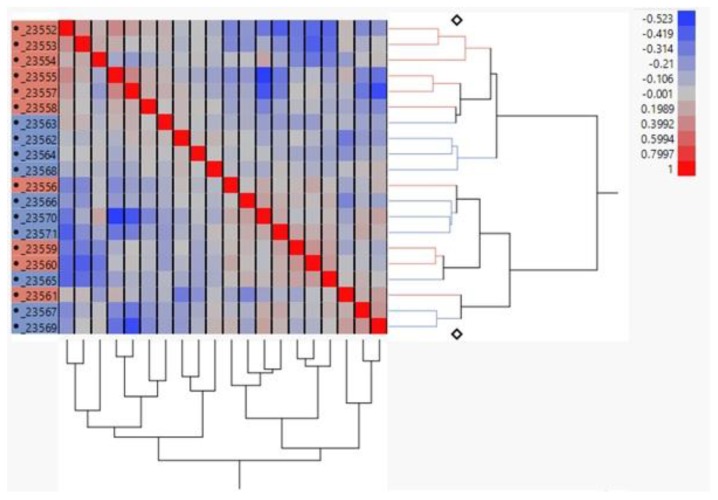
Correlation heat map of NGS-identified transcripts using upper quartile scaling normalization. The heat map represents the graphical display resulting from unsupervised clustering of the correlations between samples. The *x*- and *y*-axis are the same and the red diagonal line represents a correlation of 1. The pink labeled IDs are associated with the soybean diet while the blue labeled IDs are from the corn oil diet.

**Figure 2:**
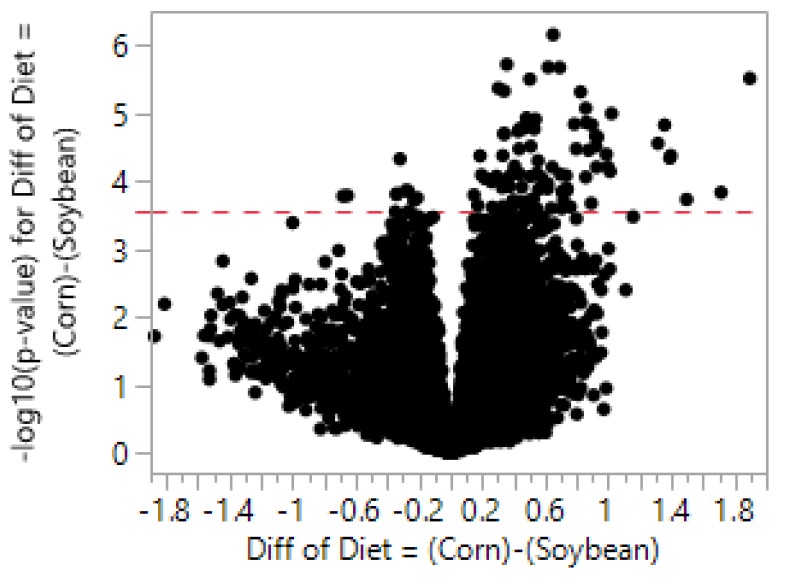
Volcano plot of NGS-identified transcripts using upper quartile scaling normalization. The *x*-axis displays the difference in log scale, and the *y*-axis plots the –log10(*p*-value) for each transcript, represented by a point. Significance threshold is graphed with a dotted red line (3.550). Points to the right of 0 and above the significance threshold are significantly increased transcripts and transcripts that are significantly decreased are to the left of 0 and above the threshold line.

Figure 1 is a representative two-way clustering heat map generated from data normalized by UQ scaling. The heat map shows that a majority of the samples clustered together according to their respective diet treatment (SO or CO). However, four SO samples (23,556, 23,559, 23,560, and 23,561) did not cluster as tightly with the other samples within the SO diet group, indicating that there may be noise in the data which is unaccounted for compared to the main effect. The data was analyzed for DEGs between the diet treatments using analysis of variance (ANOVA). Figure 2 is a volcano plot for the diet comparisons of DEGs within the samples. 100 genes were identified to be differentially expressed between the two diet groups. Altogether, 90 genes increased while 10 decreased from the CO diet with fold-changes ranging from 3.73–0.62 (Table S3).

### 3.5. Transcripts per Million (TPM) Normalization

**Figure A1:**
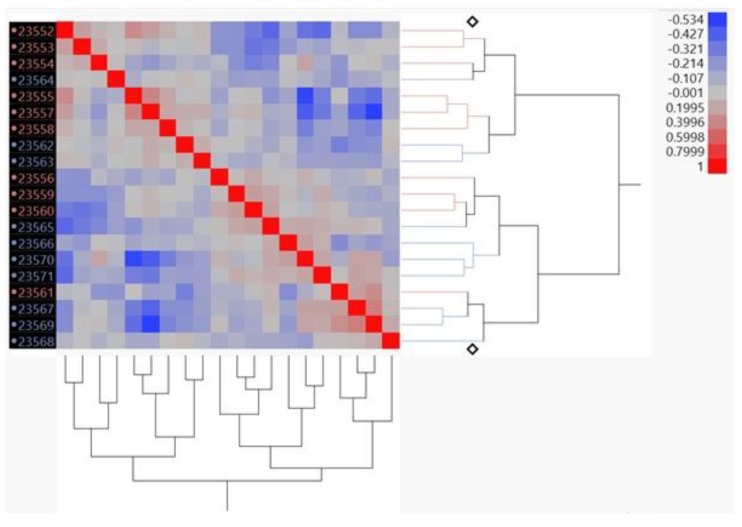
Correlation heat map using transcripts per kilobase million normalization. The heat map represents the graphical display resulting from unsupervised clustering of the correlations between samples. The *x*- and *y*-axis are the same and the red diagonal line represents a correlation of 1. The pink labeled IDs are associated with the soybean diet while the blue labeled IDs are from the corn oil diet.

**Figure A2:**
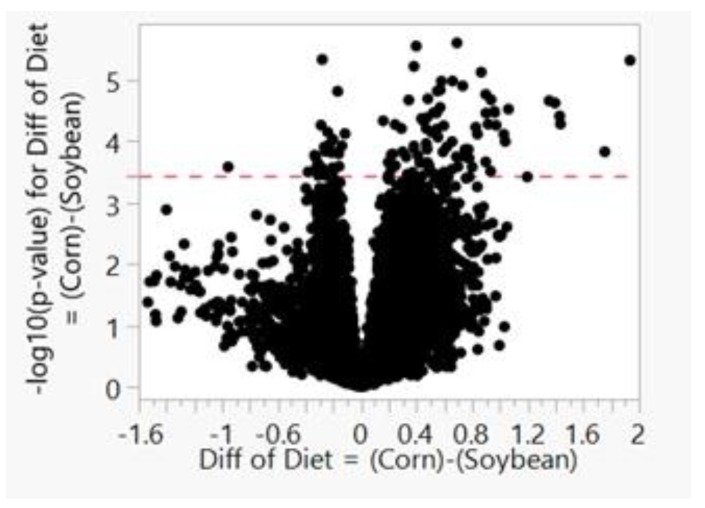
Volcano plot for upper transcripts per kilobase million normalization. The *x*-axis displays the difference in log scale, and the *y*-axis plots the –log10(*p*-value) for each transcript, represented by a point. Significance threshold is graphed with a dotted red line (3.430). Points to the right of 0 and above the significance threshold are significantly increased transcripts, and transcripts that are significantly decreased are to the left of 0 and above the threshold line.

Figure A1 in Appendix B is the corresponding two-way clustering heat map generated from data normalized by TPM. The TPM heat map is similar in clustering to the UQ scaling heat map. As described above for UQ scaling, we analyzed the data for DEGs between the diet treatments. Figure A2 is a representative volcano plot for the TPM diet comparisons of DEGs within the samples. 107 genes were identified to be differentially expressed between the two diet groups. Altogether, 81 genes increased while 26 genes decreased from the CO diet with fold-changes ranging from 3.83–0.52 (Table S3).

### 3.6. Comparison of UQ Scaling and TPM Normalization

**Figure 3:**
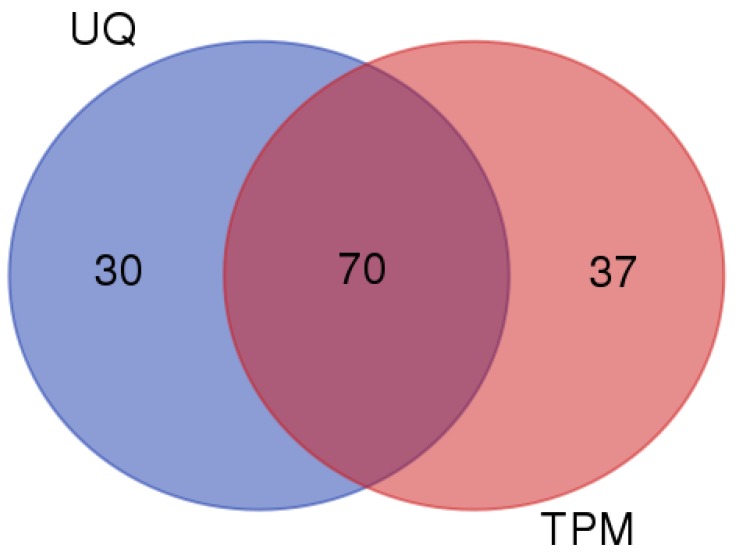
Venn diagram comparison of differentially expressed genes identified with NGS using separate data normalizations. The light pink circle represents the number of genes that were exclusively identified using TPM normalization, while the blue circle represents the number of genes that were exclusively identified using UQ scaling normalization. The dark pink oval represents the genes that overlapped between the TPM and UQ scaling normalizations.

When comparing the separate normalizations, approximately 70% of the genes overlapped that were identified as differentially expressed between the two groups (Figure 3 and Table 4). All the genes displaying at least two-fold differences were identified using both normalization methods, with the exception of *Rnr1* and *Rnr2* that were identified with UQ scaling exclusively. *Rnr1* and *Rnr2* are non-coding mitochondrial ribosomal RNAs (mt-rRNA, 12S and 16S subunits) and were filtered out during TPM normalization. Also, noteworthy was the fact that roughly 90% of DEGs identified displayed differences less than two-fold (Table S3).

### 3.7. Quantitative Real-Time PCR

We then chose to validate several (39 genes) of the NGS identified DEGs with qPCR (Table S3). We validated genes based upon the level that they were differentially expressed (i.e., top 10 genes elevated or reduced in the UQ or TPM lists) but also chose several genes based upon their involvement with energy production (*Atp5g3*, *Atp6v1h*), electron transport (*Ndufa9*, *Ndufa3*), cell proliferation or apoptosis (*Tgfa*, *Anp32b*), cell signaling or cell cycle (*Dusp1*, *Sh2b2*), neurotransmission or neurodevelopment (*Nrn1*, *Asrgl1*), and myelination (*Opalin*, *Mpzl1*). We validated NGS data with qPCR not only in the animals used for RNA-seq analysis but also in animals from a separate but identically repeated study using the same dietary treatments. The validation of gene expression in the 2nd cohort of animals provided biological replication of the data, and is not just a validation of the RNA-seq results. 

**Figure 4:**
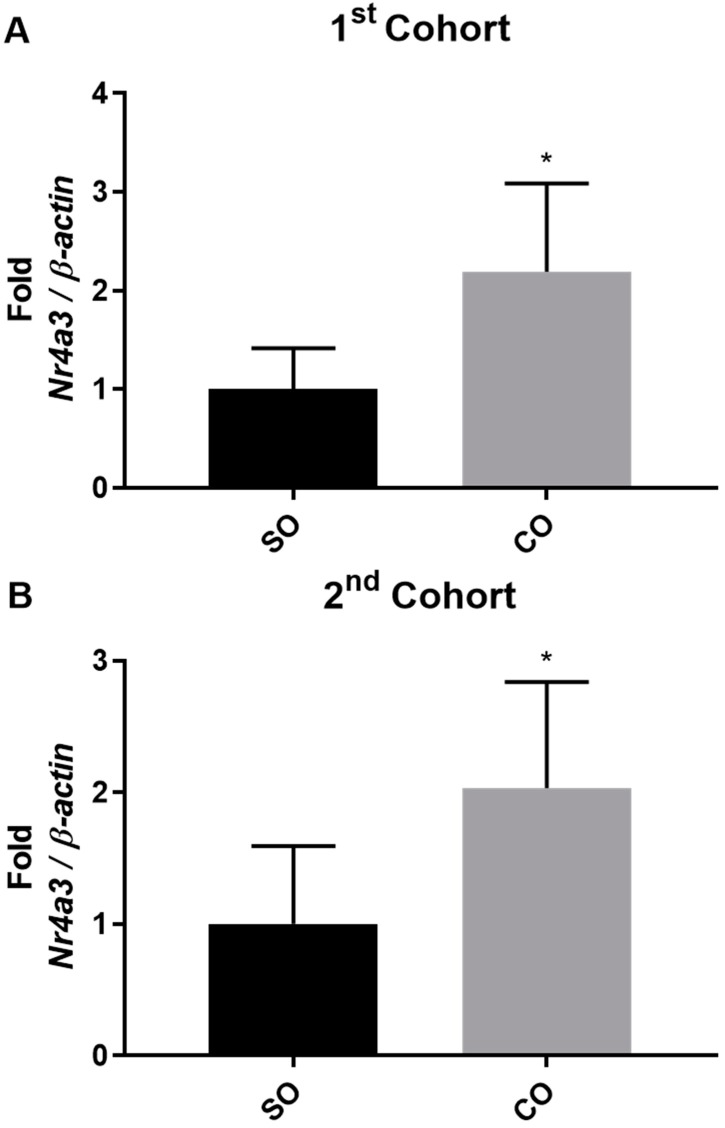
Cerebellar qPCR measurement of *Nr4a3*. Data are reported as mean ± SD, *n* = 10. * Statistical significance was assessed using Student’s *t*-test performed on the initial ΔCt values for the gene with *p* ≤ 0.05. CO: Corn oil; SO: Soybean oil; ΔCt: Change in cycle threshold normalized to β-actin.

Table S3 displays the qPCR validations for UQ scaling and TPM normalizations. Two genes (*Nr4a3* and *Smim17*) that overlapped between the normalizations, three genes (*Tmem214*, *Phactr3*, and *Amz2*) exclusively identified by UQ scaling, and one gene (*Gtf2f2*) exclusively identified by TPM were validated by qPCR in the 1st cohort of animals. However, only *Nr4a3*, whose gene product is a nuclear orphan receptor involved in transcription regulation, was confirmed in the 2nd cohort of animals to be elevated over 2-fold in the cerebellum of rats consuming the CO diet (Table S3 and Figure 4). Interestingly, eight genes (*Cpe*, *Scamp3*, *Tgfa*, *Sh2b2*, *Opalin*, *Ndufa9*, *Ndufa3*, and *Atp6v1h*) identified as being higher in CO rats by both UQ scaling and TPM normalizations were confirmed to go the opposite direction via qPCR in the 1st cohort of animals, displaying lower levels in the CO compared to SO animals (Table S3). Cerebellar *Tgfa*, whose gene product is a ligand for the epidermal growth factor receptor (EGFR), was further validated in the 2nd cohort of animals to be reduced in rats consuming CO diet.

## 4. Discussion

Undernutrition during early brain development can result in serious deficits in cognition, behavior, and motor skills that may have lasting consequences [41]. LCPUFA deficiency during brain development displays similar impairments [1]. Still, data is limited in describing what genes are altered within the transcriptome of the juvenile cerebellum in response to dietary *n*-3 PUFA deprivation.

In this study we identified 137 potential dietary *n*-3 PUFA-responsive genes within the cerebellar transcriptome of developing rats using NGS. In comparison, data obtained from the earlier cDNA microarray studies identified 102 DEGs [22], 23 DEGs [25], 24 DEGs [24], and 6 DEGs [23] in rat brains by modifying dietary *n*-3 and *n*-6 PUFA content. This correlates to roughly 0.50–0.70% (dependent on normalization) DEGs identified with NGS and the earlier studies ranged 0.20–3.40% (dependent on study). By using qPCR, we were only able to confirm that 6 of the 39 genes changed in the same direction (qualitatively consistent) with NGS data, correlating to roughly a 15% validation rate in the 1st cohort of animals. Of the 6 genes we were able to confirm in the 1st cohort, only 1 gene (*Nr4a3*) could be confirmed in the repeated study (2nd cohort). To our knowledge, brain expression of *Nr4a3* has not previously been reported to be responsive to dietary *n*-3 or *n*-6 PUFA content and was not identified as a DEG in the microarray studies listed above. It is important to note, however, that the earlier studies used different rat strains, age of rats, duration on diets, oil composition and content of *n*-3 and *n*-6 PUFA within diets, whole brain or hippocampal tissue, and RNA samples were pooled 8–12 animals/group. So, a fair comparison between the earlier studies and the current study cannot be made.

The majority of the cerebellar DEG fold-changes in this study were <2-fold. This finding is consistent with a previous RNA microarray study examining DEGs within the cerebral cortex of young baboons that were fed diets that differed in amounts of DHA and AA for 12 weeks [42]. Furthermore, a more recent microarray study that analyzed modifications in the mouse brain transcriptome by altering dietary *n*-3 to *n*-6 levels for 20 weeks, identified roughly 1000 genes of interest but less than 8% were >2-fold changes [43]. These microarray studies identified more potential *n*-3 and *n*-6 PUFA responsive genes within the brain; however, the extent to which the DEGs were modified was low. While there are several important experimental design differences compared to our study, namely dietary *n*-3 and *n*-6 LCPUFA content and duration on diets, the LCPUFA-dependent modifications within the brain transcriptome was relatively small, which is consistent with our results. These results are quite different from a recent study analyzing effects of dietary *n*-3 and *n*-6 PUFA within the pig liver transcriptome that report over 3500 DEGs with average fold-changes of 9.4 [44]. These previous data, in addition to our data, indicate that the brain transcriptome is tightly regulated with regard to dietary *n*-3 PUFA.

The most prominent finding in this study was identifying and confirming the elevated expression of *Nr4a3* from CO in both cohorts of animals. *Nr4a3* (gene product—NOR1) is a transcriptional activator known for playing important roles in cell survival and apoptosis [45,46]. The *Nr4a* gene family regulates the neuroendocrine system which is responsible for energy utilization and metabolism [47]. In the brain, *Nr4a3* is capable of providing neuroprotection and promoting neuronal survival upon excitotoxic and oxidative stress generated from kainic acid, a glutamate analog, in the hippocampus of mice [48,49]. Moreover, *Nr4a3* and other related *Nr4a* gene members are elevated after learning and memory formation in mice and their activation is associated with increased synaptic plasticity [50,51]. Previous studies using mouse skeletal muscle cells and tissue have shown that *Nr4a3* plays a role in regulating genes involved with fatty acid utilization and is inducible by the beta-adrenergic receptor agonist isoprenaline [52,53]. In beta cells of the pancreas, *Nr4a3* is inducible by FAs (palmitate) and can modulate secretion of insulin and expression of the *Ins1* and *Ins2* genes [54]. From our results, it is not clear if the modified level of *Nr4a3* within the cerebellum is an adaptive response to the stress imposed by dietary *n*-3 PUFA deprivation, or if dietary *n*-3 PUFA deprivation is causing cognitive disturbances. More work is needed to answer these questions.

Reductions in DHA are observed in the rat brain from trans-generational dietary *n*-3 PUFA deprivation [55,56]. Under natural conditions (non-trans-generationally deprived of dietary *n*-3 PUFA), the postnatal rat brain is effective in accumulating DHA required for growth and is effective in limiting DHA loss during times of dietary *n*-3 deprivation [57]. The juvenile *n*-3 PUFA deficient rat model used in this study is efficient in depleting *n*-3 PUFA from livers and altering the brain FA content [29,30]. In prior studies, we have observed small (~10%) reductions in cerebellar levels of DHA and increases in DPA*n*-6, AA, and DTA using the CO diet in postnatal rats [29,30]. The cerebellar FA content reported here is in good agreement with our earlier studies, with the exception of the DHA reduction in the brains of young rats fed CO (~0.8 mol% ALA) for four weeks. This finding is not unexpected, since the DHA reduction demonstrated previously was minor and ~15 weeks is needed to have more prominent reductions in brain DHA when rats are fed diets utilizing this percentage of ALA [58]. 

A longer postnatal duration of dietary n-3 PUFA deprivation or initiating deprivation prenatally may have resulted in a more pronounced effect to the LCPUFA content within the cerebellum and reduced DHA more effectively. More prominent alterations to the cerebellar LCPUFA composition could have resulted in greater fold changes to the DEGs identified in this study or generated an entirely different list of DEGs. Additional studies are needed to assess the possible health consequences associated with the cerebellar LCPUFA-dependent modification of *Nr4a3* expression during development and whether these effects are reversible.

This study has limitations. The cellular heterogeneity of the postnatal cerebellum coupled to its dynamic growth and remodeling during this period of time add complexity in measuring DEGs within this important developing tissue. A majority of the DEGS identified displayed only minor changes (<2-fold) and from a statistical standpoint, there is an abundance of NGS data (*n* = 10/group). This results in increased precision around the estimated means and potentially Type II statistical errors. However, we performed qPCR validations on a 2nd cohort of animals to provide biological replication and reduce the impacts of statistical anomalies.

## 5. Conclusions

This study provides insight about the transcriptomic modifications within the juvenile rat cerebellum imposed by changing *n*-3 PUFA content of the diet. Several potential *n*-3 PUFA responsive genes were identified, however, only *Nr4a3* was confirmed to be elevated over 2-fold from the CO diet. In the brain, *Nr4a3* provides neuroprotection but is also involved in cognition and memory formation. This work provides a foundation for developing future studies to better understand what mechanisms are involved with LCPUFA regulation of brain *Nr4a3* expression. Additional research is needed to clarify whether synaptic plasticity, learning, or memory formation is affected in these developing animals given the neurodevelopmental importance of LCPUFA along with the important functions of *Nr4a3* within the brain.

## Figures and Tables

**Table 1:** Body composition in growing male rats.

	1st Cohort			2nd Cohort		
Endpoint	SO	CO	*p*	SO	CO	*p*
Body Mass (g)						
Begin	53.7 ± 2.2	54.8 ± 2.7	0.32	57.1 ± 4.0	57.4 ± 4.1	0.85
End	224.9 ± 7.7	235.9 ± 10.2	0.01	253.2 ± 11.2	253.8 ± 15.5	0.92
Liver Mass (g)	6.6 ± 0.4	7.3 ± 0.7	<0.01	7.4 ± 0.4	7.3 ± 0.5	0.59
Brain Mass (g)	1.6 ± 0.1	1.6 ± 0.1	0.27	1.7 ± 0.1	1.7 ± 0.1	0.74
Brain/Body (%)	0.69 ± 0.04	0.67 ± 0.05	0.43	0.65 ± 0.03	0.65 ± 0.04	0.93

Data are reported as mean ± SD, *n* = 10. Student’s *t*-test was performed for each parameter. Bold indicates significance, *p* < 0.05. CO: Corn oil; ER: Energy restriction; SO: Soybean oil.

**Table 2:** Fatty acid concentration in the cerebellum of juvenile male rats.

Tissue Concentration, µmol/g			
FAs	SO	CO	*p*
16:0	17.23 ± 2.16	19.08 ± 1.89	0.06
18:0	14.62 ± 1.92	15.62 ± 1.47	0.21
20:0	0.35 ± 0.06	0.33 ± 0.04	0.53
22:0	0.22 ± 0.03	0.22 ± 0.03	0.94
24:0	0.22 ± 0.05	0.23 ± 0.04	0.97
16:1*n*-7	0.30 ± 0.03	0.30 ± 0.03	0.74
18:1*n*-7	3.37 ± 0.44	3.60 ± 0.35	0.22
18:1*n*-9	14.19 ± 2.03	14.76 ± 1.35	0.47
20:1*n*-9	1.50 ± 0.24	1.42 ± 0.16	0.42
22:1*n*-9	0.18 ± 0.02	0.17 ± 0.03	0.37
18:2*n*-6	1.29 ± 0.19	1.23 ± 0.13	0.44
20:2*n*-6	0.35 ± 0.06	0.33 ± 0.03	0.51
20:3*n*-6	0.41 ± 0.07	0.40 ± 0.03	0.42
20:4*n*-6 (AA)	6.11 ± 0.81	6.96 ± 0.61	**0.02**
22:4*n*-6 (DTA)	1.92 ± 0.28	2.33 ± 0.19	**<0.01**
22:5*n*-6 (DPAn6)	0.27 ± 0.03	1.13 ± 0.17	**<0.01**
*n*-6 LCPUFAs ^1^	9.06 ± 1.22	11.15 ± 0.93	**<0.01**
22:5*n*-3	0.31 ± 0.12	0.27 ± 0.11	0.42
22:6*n*-3 (DHA)	10.13 ± 1.33	10.01 ± 0.85	0.81
*n*-3 LCPUFAs ^2^	10.44 ± 1.39	10.27 ± 0.85	0.75

Data are reported as mean ± SD, *n* = 10. Student’s *t*-test was performed for each parameter. Bold indicates significance, *p* < 0.05. ^1^ *n*-6 LCPUFAs sums all *n*-6 PUFAs except 18:2*n*-6. ^2^ *n*-3 LCPUFAs sums all *n*-3 PUFAs. CO: corn oil; FA: Fatty acid; LCPUFA: Long-chain polyunsaturated fatty acid; SO: Soybean oil.

**Table 3:** Next-generation sequencing data.

Group	ID	Paired Reads	Uniquely Mapping	%	Annotated Transcripts
SO	23552	16,672,271	12,849,214	77.1	20,399
	23553	22,682,731	19,036,095	83.9	20,944
	23554	14,456,057	11,930,561	82.5	19,577
	23555	29,567,630	19,869,689	67.2	21,578
	23556	28,959,204	23,777,578	82.1	21,562
	23557	17,119,385	13,192,970	77.1	20,715
	23558	11,037,221	8,945,862	81.1	19,354
	23559	36,432,242	30,316,383	83.2	22,242
	23560	33,211,308	27,063,716	81.5	21,985
	23561	49,759,399	36,660,886	73.7	22,787
CO	23562	15,976,576	13,229,505	82.8	20,080
	23563	24,312,065	18,465,257	76.0	21,272
	23564	16,002,390	13,155,503	82.2	20,556
	23565	48,831,450	38,802,478	79.5	23,180
	23566	23,814,884	19,463,164	81.7	21,361
	23568	17,616,302	14,507,861	82.4	20,296
	23569	54,876,507	44,754,936	81.6	22,953
	23570	54,528,411	45,878,644	84.1	22,850
	23571	31,881,735	26,108,279	81.9	21,694
	23567	35,021,051	28,774,451	82.2	21,789

% is the percentage of paired reads that uniquely mapped to the rat genome (Rnor_6.0). CO: Corn oil; ID: sample identification number; SO: Soybean oil.

**Table 4:** Differentially Expressed Genes Identified Specific to Data Normalization.

Normalization	Total	Genes
UQ & TPM	70	*Ube2l3, Atp6v1h, Dusp1, Trnp1, Dbndd2, Scpep1, Tmem42, Thy1, Tlk1, Phactr3, Ndufa9, Gmfb, Klhl11, C2cd3, Opalin, Tmem231, Ptafr, Gstt3, Nrn1, Elovl2, Atp5g3, Tmem206, March8, S100b, Fam13c, Tbc1d15, Hsp90ab1, Ftsj1, Asrgl1, Htra1, Gga3, Pitrm1, Rnasek, Rpl7l1, Tgfa, Ifngr1, Sox8, Scamp3, Pgap2, Tspan1, Elp5, Ldoc1, Anp32b, Yars, Cpe, Klhl2, Fam220a, Mpzl1, Zfp212, LOC314140, Mrpl40, Sh2b2, Nxph4, Idh3a, Sec62, Ankrd40, Stmn2, Nr4a3, Bfar, Atp6v0c, Rps16, Hsbp1, LOC100911248, Ndufa3, Lix1l, Zfp449, Ralbp1, Dnajc7, Srfbp1, Smim17*
UQ	30	*LOC108351039, LOC103692551, Pik3cb, Ttc33, Socs5, LOC103690204, Prepl, Rnr1, Rcbtb2, Ppp3r1, Wee1, Noa1, Mfsd11, Gatm, Gmcl1, Rnr2, Pde4b, Tmem214, Meis3, Errfi1, Trnm, Mbtd1, Cnih1, Rab7a, Vac14, Trap1, Clcn4, Amz2, Ywhae, Aida*
TPM	37	*Nlrp3, Golga5, Ube2j2, Ica1, Snrpa1, Gtf2f2, Abcf2, Uvrag, Vps45, Stim2, Metap2, Kpna1, Nkain4, Nutf2, Rundc1, Ppp1r8, Tnpo3, Pithd1, Osbpl2, Samd10, Fbxw4, Cul1, Ptpmt1, Tmem184c, Cdc34, Cnrip1, Atp5a1, Metap1, Ppcs, Vdac2, Pold3, Cetn2, Mrpl9, Ndufb7, Qrich2, Pex3, Gadd45gip1*

UQ: Upper quartile; TPM: Transcripts per million.

## Supplementary Materials

The following are available online at http://www.mdpi.com/2072-6643/11/2/407/s1, Table S1: Genes quantified using upper quartile scaling, Table S2: Genes quantified using transcripts per million, Table S3: NGS DEGs with qPCR validation in two separate animal cohorts.

## Appendix A

**Table A1:** RNA quality used in analysis.

Group	Sample	RQN
SO	23,552	8.1
	23,553	8.2
	23,554	7.0
	23,555	8.2
	23,556	*
	23,557	8.1
	23,558	8.2
	23,559	8.2
	23,560	8.4
	23,561	8.3
CO	23,562	8.1
	23,563	7.7
	23,564	8.1
	23,565	8.3
	23,566	7.9
	23,568	8.2
	23,569	7.9
	23,570	6.6
	23,571	8.2
	23,567	8.6

* Instrument error. CO: Corn oil; RQN: RNA quality number; SO: Soybean oil.

**Table A2:** qPCR primer sequences.

Gene	Forward	Reverse
*Meis3*	GCTCCTCTGACTCCTTCAATG	ATCCTCGATCACCAGGTCTAT
*Tmem214*	GCTGCAAGAGCTGGATAAGA	CTTTGGTGAGGTTGGGATACA
*Ralbp1*	ACCAAGATTGCACAGGAGATAG	CTGGGCAAGGAGGATATTTATGA
*Tmem42*	CCCTGATGTGGACCTTCTTTAG	CTAAGATGGCCGAGCAAAGTA
*Noa1*	CGGGCTATGGAGTTGAAGAA	AGGAGTCGGGTTGCAAATAG
*Phactr3*	CCGTGGAAGAACTGGAAAGA	CTTGTCTGCTCTCCTGTCATAG
*Trap1*	GCTGACCAGCTTATCAGACTAC	CTCCACAGAGATCAGCTTCTTC
*Amz2*	CTGCGACTAAGTGCTCCTTTA	CTTCACGCTGCCTTCATATTTC
*Smim17*	CAGCTGTGACAGTGATGAGAA	TGGAGCATCATGGGCATC
*Scamp3*	CAGGCACTCGACAGAACAA	GACAAAGGAGCAGGGAGTAAA
*Cpe*	GAGTGGTACTGCTCACGAATAC	GGTCACGGACAAACCCTTTA
*Atp6v0c*	CTTATCGCTAACTCCCTGACTG	AAGCACCTCCGCAAAGAT
*Gstt3*	CAGCATTACACTGATGCCTTTG	GTCAGGTGCCTTGTACTTTCT
*Nxph4*	CACCAAGAGAAAGCCATCCA	CAGCGAGAACTTGAGGGTATG
*Ldoc1*	TGCGAATACTGGTGTGTGAG	ACAGGAGGCTGATCAAGAATG
*Nr4a3*	GCAGAGCCTGAACCTTGATA	CATAGCTCCTCCACTCTCTTTG
*LOC100911248*	TGTCTCAGCACCCAACTTATC	CAACGTAAGGGACAGGAATCA
*Rps16*	CAAGTTACTGGAGCCTGTTCTG	CCGATCGTACTGGATGAGGATA
*Qrich2*	GGCCTTGATGGACACATCTATAA	CTGGAGCTTAGCCTTGTTGT
*Vps45*	CCCTGGACCATCTCATCAAAG	CATAGGTGGCTCCTCCAATAAC
*Cdc34*	GACCTCTTCTACGACGACTACTA	GCCAGAGTCATCCTCTTCATC
*mrpl9*	GTACTGGTGTGATGTGACAGTAA	GGCTAACCAGTGCTTGTATCT
*Gadd45gip1*	GCCCGATTCCAGGAACTATTAC	GCAATTCGAGCCTCCTTCTT
*Fbxw4*	TGCTTACACACCATCCAGAC	TGAGAAGTGCCCACAACAA
*Gtf2f2*	CCTGTGGGATACCTGAAAGAAA	CCTCTGTTTGGTAATGCCTGTA
*Pithd1*	GGGCAATGTCAAGCTCAAAG	GTCTGGCTCCCTTTCTGTATC
*Ppcs*	CAGACCCGGACATCATCATTAG	GTCCTCAGACAGCAGTAACTTT
*Sh2b2*	GTGGTGTCAGTGAGAGCAATAA	GCCGTGACCATTCAGAGAAA
*Tgfa*	CCAGATTCCCACACTCAGTATT	GGATCAGCACACAGGTGATAA
*Anp32b*	GTCACTAACCGGAGTGATTACC	CATCTTCTTCCTCCTCACCATC
*Dusp1*	CTAACCACTTTGAGGGTCACTAC	GATAATACTCCGCCTCTGCTTC
*Nrn1*	GGGCGAAAGATATGTGGGATAA	CTGCCGCAGAGTTCGAATAA
*Asrgl1*	CAAGAAGCACCTGGAGAAAGA	GGCCAGATTCACCTTCAGAATA
*Opalin*	GCTGGTAGCCTTGCTGTTTA	CCACCAAAGACCTCTTCTTCTC
*Ndufa9*	TCCGCTTTCAGGTTGTTAGAG	GCATCCCACTCCAGAAAGATAA
*Ndufa3*	AAAGAACCACAACTCCCAGAG	ACAGGTTCTTGAGCCATTCC
*Atp5g3*	GGAGAGGGCTCTACGGTATTTA	GAGACCCATAGCTTCAGACAAG
*Mpzl1*	GGCTCCATGTGGTGGAAATA	GTGGTCTAACTGTGCGTATATGA
*Atp6v1h*	CCAAGAGTATGCTCTGGCTATG	CCACCGAGCTGCTCAATAA
*β-actin*	GTGTGGATTGGTGGCTCTATC	CAGTCCGCCTAGAAGCATTT
